# Social Isolation Alters Social and Mating Behavior in the R451C Neuroligin Mouse Model of Autism

**DOI:** 10.1155/2017/8361290

**Published:** 2017-01-31

**Authors:** E. L. Burrows, A. F. Eastwood, C. May, S. C. Kolbe, T. Hill, N. M. McLachlan, L. Churilov, A. J. Hannan

**Affiliations:** ^1^Florey Institute of Neuroscience and Mental Health, Melbourne Brain Centre, University of Melbourne, Parkville, VIC 3010, Australia; ^2^Department of Anatomy and Neuroscience, University of Melbourne, Parkville, VIC 3010, Australia; ^3^Psychological Sciences, University of Melbourne, Parkville, VIC 3010, Australia; ^4^Florey Institute of Neuroscience and Mental Health, 245 Burgundy St, Heidelberg, VIC 3084, Australia

## Abstract

Autism spectrum disorder (ASD) is a neurodevelopmental disorder typified by impaired social communication and restrictive and repetitive behaviors. Mice serve as an ideal candidate organism for studying the neural mechanisms that subserve these symptoms. The Neuroligin-3 (NL3) mouse, expressing a R451C mutation discovered in two Swedish brothers with ASD, exhibits impaired social interactions and heightened aggressive behavior towards male mice. Social interactions with female mice have not been characterized and in the present study were assessed in male NL3^R451C^ and WT mice. Mice were housed in social and isolation conditions to test for isolation-induced increases in social interaction. Tests were repeated to investigate potential differences in interaction in naïve and experienced mice. We identified heightened interest in mating and atypical aggressive behavior in NL3^R451C^ mice. NL3^R451C^ mice exhibited normal social interaction with WT females, indicating that abnormal aggressive behavior towards females is not due to altered motivation to engage. Social isolation rearing heightened interest in social behavior in all mice. Isolation housing selectively modulated the response to female pheromones in NL3^R451C^ mice. This study is the first to show altered mating behavior in the NL3^R451C^ mouse and has provided new insights into the aggressive phenotype in this model.

## 1. Introduction

Autism spectrum disorder (ASD) is a neurodevelopmental disorder characterized by impaired social communication and repetitive and restrictive behavior [1]. Reflecting the diverse clinical presentation, ASDs are no longer thought to have a single causal factor. ASDs have a high heritability and 10–25% of individuals with the condition possess an associated genetic disorder [2]. This genetic link is further strengthened by studies that demonstrate a familial concordance ranging from 60 to 90% for ASDs between monozygotic twins [3, 4]. Of the associated genetic variants and mutations, many affect proteins involved in synapse function and development [5]. In particular, mutations in single genes that encode cell-adhesion molecules such as the neuroligin/neurexin complexes have been identified [6–10]. Neuroligins are proteins localized to the postsynaptic membrane [11, 12] and function as ligands to presynaptic neurexins, forming dynamic transsynaptic neurexin/neuroligin complexes, which putatively subserve synaptic formation [12–15] and function [16–18]. Disruption to the regulation of these pivotal synaptic proteins may provide insight into dysregulated synaptic mechanisms in ASD [11, 19–21]. Notwithstanding these strong genetic bases, there is evidence for a role of environmental modulation in the etiology of ASDs. Discrete modules of coexpressed ASD-associated genes specifically enriched in high-throughput RNA-sequencing but not GWAS implicate a nongenetic causative factor and potential interplay between genetic predisposition and the environment [22].

The identification of many genetic mutations associated with ASDs prompts the use of mouse models to further our understanding of how these may lead to the underlying physiological mechanisms. Aberrant reciprocal social interactions can be probed in mouse models using assays that measure tendency to spend time with an unfamiliar mouse [20, 23]. The NL3 R451C mutation, discovered in two Swedish brothers with ASDs [6], results in only 10% of the functional protein being incorporated into the synaptic architecture [10] and affects the binding of NL3 to its presynaptic neurexin ligand [24]. NL3^R451C^ mice show a diverse range of behavioral abnormalities, including altered social interaction, restrictive and repetitive behaviors, and synaptic dysfunction as shown by increased cortical inhibition together with enhanced hippocampal excitation in brain slices [25–32]. Inconsistencies between investigations of social interaction in NL3^R451C^ mice have been reported [27–29]. These discrepancies have been said to be largely due to different genetic backgrounds, experimental conditions, and tests conducted by different laboratories [27, 33]. Heightened aggression towards younger sexually mature male mice has also been reported using a resident-intruder assay where the animals were permitted free interaction [31]. No aggression has been noted during free interaction juvenile social interaction testing, suggesting that the heightened aggression expressed in the adult NL3^R451C^ mouse could be territorial in nature [27, 31]. Aggression in mice is a robust, innate, social behavior and serves to assist the acquisition of social ranking and resources from the environment, including female mates. Alterations in this behavior add to the understanding of the NL3^R451C^ mouse social phenotype. Overlapping neural circuits control aggression and mating behavior in mice; however, it is not known if NL3^R451C^ mice show any differences in social or mating behavior towards female mice [34, 35]. In order to determine whether the NL3 R451C mutation impacts social and mating behavior in male-female diads, we assessed interactions with female mice in male mutant and WT mice. The aim of the present study was also to address how context influences the behavior of adult male mice during social interaction. Mice were housed in social isolation from weaning to probe if this increased social interactions differentially in mice. Isolation housing has been previously shown to potentiate social interaction in mice [36]. Furthermore, tests were repeated to investigate whether experience altered social behavior between groups.

## 2. Methods

### 2.1. Animals and Housing

B6;129-*Nlgn*3^*tm1Sud*^/J mice were obtained from Jackson Laboratories (Bar Harbor, Maine, USA) and backcrossed beyond generation F10 on a C57BL6 background. NL3^R451C^ and WT animals were derived by mating heterozygous females with NL3^R451C^ males, which produced 50 : 50 WT and NL3^R451C^ male offspring (Y/+ and Y/R451C) that were genotyped as previously described [28]. All mice were socially housed until weaning at postnatal day 28 in conventional open-top cages (31 × 16 × 10 cm) with basic nesting materials (pine bedding and tissue paper) maintained at a constant temperature (22 ± 1°C). After weaning, all mice were transitioned from a standard 12-hour light-dark cycle (light: 07:00–19:00) to another 12-hour reverse-cycle room (light: 19:00–07:00) over three days (4-hour cycle shift per day). Food and water were available ad libitum. C57Bl/6J female mice were housed in groups of 5-6 individuals. At weaning, mice were pseudorandomly allocated to mixed housing (3-4 individuals) or social isolation, ensuring equal WT and NL3^R451C^ mice in each mixed housing condition and that litters were spread over all conditions. All socially housed mice were individually housed following the first Male-Female Social Interaction Test (MFSIT; aged 13–19 weeks), to avoid excessive aggression, previously reported in NL3^R451C^ adult male mice [31]. Experiments occurred between 08:00 and 18:00, during the dark cycle under red light (4 lux) at 55.0% humidity; mice were habituated to experimental rooms (22 ± 1°C) for at least 30 minutes prior to testing, from which all strong odors were eliminated. All experiments were approved by the Florey Institute of Neuroscience and Mental Health Animal Ethics Committee.

### 2.2. Estrus Cycle Determination

Adult wild-type C57Bl/6J female mice were pap-smeared on the morning of each experimental day in order to determine the respective stage in the murine estrus cycle. Only those determined to be in estrus on the same experimental day were used either for urine collection or as a stimulus mouse. Animals were held by the base of their tails, with their hind limbs raised to evert their genital region and vaginal epithelial and blood cells were collected using a cotton-tipped applicator and smeared on the surface of a sterile glass microscopy slide and allowed to dry. Estrus phase was determined by staining cells using Thermo Scientific™ Shandon™ Kwik-Diff™ staining kit (Thermo Fisher Scientific Inc., USA).

### 2.3. Female Urine Sniffing Test (FUST)

The Female Urine Sniffing Test has been described previously [37]. In brief, for at least one hour prior to the test, 8–10-week-old mice were habituated to a sterile cotton-tipped applicator suspended from the ceiling of a clean cage in the reverse light cycle dim red light illumination (4 lux). Urine from C57Bl/6J estrus female mice was thawed from a −80°C freezer to room temperature and combined into a single vial. The same urine combination was used within each experimental day. Urine (10 *μ*L) was pipetted onto another sterile cotton-tipped applicator and suspended into the cage for 3 minutes. Latency and duration of sniffing were recorded using a digital camera (Panasonic, Secaucus, NJ, USA), positioned 30 cm from the cage, and were scored manually post hoc by a single independent observer blinded to genotype and housing condition.

### 2.4. Male-Female Social Interaction Test (MFSIT)

Social behavior between socially and isolation-housed male mice, aged 14–20 weeks of age, towards a novel sexually mature female was assessed using a previously described protocol [38]. Each male experienced the MFSIT twice, one week apart, to probe for the effect of sexual experience. Females (in estrus, determined same day of testing) were pseudorandomly paired with the subject male, ensuring that each dyadic interaction was novel and that each female was paired with only one male per day. For at least one hour prior to the test, all mice were habituated to the experimental room. Male mice were additionally habituated to clean, transparent Perspex open-top cages (31 × 16 × 15 cm) with fresh, odorless pine litter. In Phase 1, the stimulus female was placed into the cage with the male for a 5 min period of free interaction. Following this, the female was removed and placed into a separate clean standard open-top cage. After 3 mins, the same female was then recoupled with the male for a second 5 min bout of free interaction (Phase 2). The test was repeated one week later where males were paired with a different female. Behaviors were recorded using a digital visual camera (Panasonic, Secaucus, NJ, USA), positioned 30 cm above the cage. Sniffing, stalking, mounting, and attacking behaviors (previously defined in [31, 39]) were scored by a single observer blinded to genotype and housing condition post hoc using a key-sensitive timer program custom written in MATLAB®. For each behavior, latency, number of bouts, and total duration were analyzed. Sniffing behavior was recorded when mice made contact with the female with their nose and were stationary. Stalking was defined as slow deliberate chasing behavior. 5 trials were chosen at random and scored by an independent observer. High concordance between the behaviors scored was seen (Supplementary Figure  1 available online at https://doi.org/10.1155/2017/8361290).

### 2.5. Statistical Analyses

FUST data was normally distributed and variance comparable and two-way ANOVA followed by Bonferroni post hoc tests was applied. MFSIT data was not normally distributed and random effect regression models were applied to estimate the effect size of each behavioral measure. Animals were repeatedly tested over 2 phases and over 2 weeks; thus these observations are correlated within a given animal. In all analyses, phase, episode, housing, and gene were used as independent variables. Two-sided *p* values were reported together with appropriate effect size estimates and 95% confidence intervals (95% CI) to indicate the precision. Latency describes the time to a behavior and may be censored (e.g., when an animal does not attack during the 300 sec observation period). A shared frailty Cox regression model was used to estimate the treatment effect size, measured as the hazard ratio of the first sniff/mount/attack occurring at any time over the 300 sec observation period. A negative binomial regression model was used to estimate the differences in number of mounting episodes, measured as the ratio of expected number of mounts. Clustered median regressions were applied to the duration data of those animals engaged in the specific behavior. Results for MISFIT data are graphically presented as a box and whiskers plot showing the 25th to the 75th percentile and the minimum to maximum of the data range and median shown by a line. Data from FUST are shown as mean ± SEM. Significance was evaluated at *p* < 0.05. Statistical analyses were performed with STATA v13IC (StataCorp, College Station, TX, USA) and IBM SPSS Statistics v22 (IBM Corp, Armonk, NY) software.

## 3. Results

### 3.1. Social Isolation Potentiates Social Behavior in Both NL3 and WT Mice

**Figure 1:**
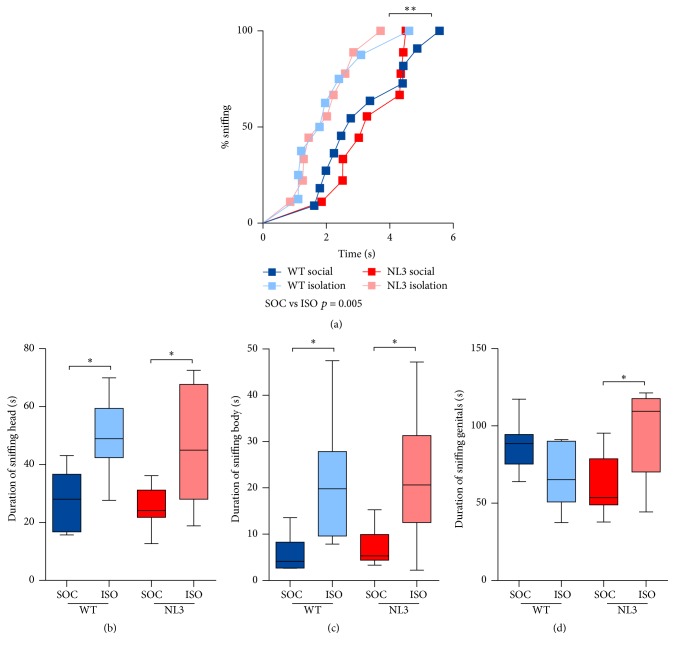
Isolation housing increased sociability in WT and NL3 mice. (a) Latency to sniff, (b) sniffing head of female, (c) sniffing body, and (d) sniffing genital region. Values are an average of 4 tests (2 phases, 2 weeks) and data in (b–d) are displayed as boxplots with median plus the 25th and 75th percentiles. Whiskers represent the minimum and maximum values. SOC = socially housed animals (WT: *n* = 10; NL3 = 10); ISO = isolation-housed (WT: *n* = 9; NL3 = 9) animals. ^*∗*^*p* < 0.05 and ^*∗∗*^*p* < 0.01.

NL3^R451C^ and WT mice were assessed for social and mating behavior towards a female mouse, over two phases (first and second exposure, following a 3-minute separation) and across two weeks (naïve and experienced, one week apart). No group differences were seen in latency to sniff, with all mice making contact with the female in under 6 seconds regardless of exposure or week tested (Figure 1(a)). Mice housed in isolation showed increased interest in interacting with the female, spending more time sniffing their head (Figure 1(b); median difference in time between social and isolation housing = 19.01; *p* = 0.005; 95% CI: 5.9, 32.12) and body (Figure 1(c); median difference in time between social and isolation housing = 13.34; *p* < 0.001; 95% CI: 6.02, 20.65) compared to socially housed animals. Isolation housing had a selective effect on time spent sniffing the genital region of the female mouse in NL3 mice only (Figure 1(c); median regression, gene*∗*housing interaction: *p* < 0.001). NL3 mice were more likely to sniff the genital region of the female mouse when they were housed in isolation (Figure 1(c); median difference in time between social and isolation housing for NL3 only = 43.0; *p* = 0.006; 95% CI: 13.09, 72.92).

**Figure 2:**
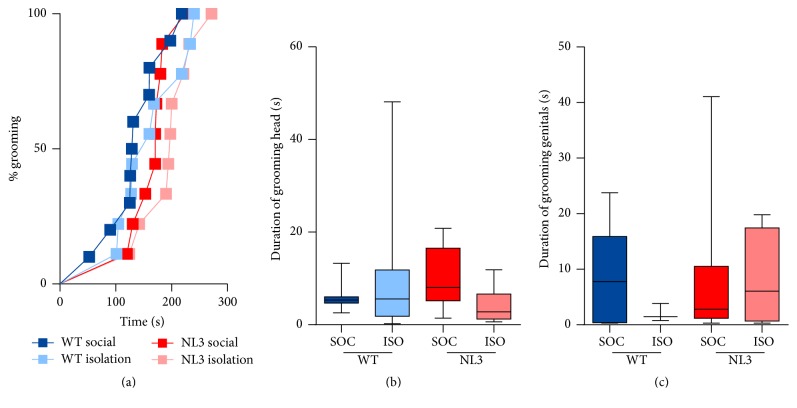
Self-grooming behavior in NL3 and WT mice. (a) Latency to groom, (b) time spent grooming head/body, and (c) genitals. Values are an average of 4 tests (2 phases, 2 weeks) and data in (b-c) are displayed as boxplots with median plus the 25th and 75th percentiles. Whiskers represent the minimum and maximum values. SOC = socially housed animals (WT: *n* = 10; NL3 = 10); ISO = isolation-housed (WT: *n* = 9; NL3 = 9) animals.

Mice were scored for latency to groom (Figure 2(a)) and, provided they groomed within the session, they were scored for time spent grooming their head and body (Figure 2(b)) and genitals (Figure 2(c)). No differences were seen between WT and NL3 mice and no effect of housing on any measure was evident indicating that any differences seen in dyadic interactions were not due to time spent self-grooming.

### 3.2. NL3 Mice Show Altered Mating Behavior and Isolation Housing Does Not Modify Behavior

**Figure 3:**
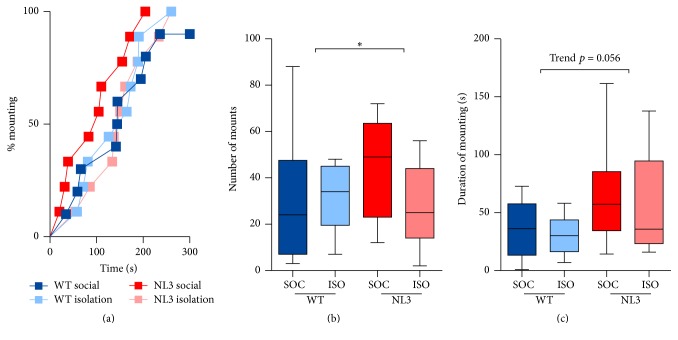
NL3 mice show heightened interest in mating compared to WT mice. (a) Time to first mount, (b) number of mounts, and (c) duration of mounting. Values are an average of 4 tests (2 phases, 2 weeks) and data in (b-c) are displayed as boxplots with median plus the 25th and 75th percentiles. Whiskers represent the minimum and maximum values. SOC = socially housed animals (WT: *n* = 10; NL3 = 10); ISO = isolation-housed (WT: *n* = 9; NL3 = 9) animals. ^*∗*^*p* < 0.05.

NL3^R451C^ and WT mice did not show any difference in latency to mount (Figure 3(a)). All mice were quicker to mount after the period of separation (Figure 3(a); hazard ratio of first mount in second phase compared to first = 3.09; *p* < 0.001; 95% CI: 1.99, 4.81) and slower when the test was repeated a week later (Figure 3(a); hazard ratio of first mount in second week compared to first = 0.51; *p* = 0.004; 95% CI: 0.33, 0.80). NL3^R451C^ mice mounted a greater number of times compared to WT mice (Figure 3(b); ratio of expected number of mounts between WT and NL3 mice = 1.95; *p* = 0.004; 95% CI: 1.24, 3.06). A lack of interaction between housing and genotype meant that we were unable to ascertain if this effect was specific to one condition. A trend for NL3^R451C^ mice to spend longer mounting the female mouse was also observed (Figure 3(c); median difference in time between WT and NL3 mice = 39.42; *p* = 0.056; 95% CI: −1.05, 79.89). Duration mounting increased in all mice when they were exposed to the same female after a brief period of separation (Supplementary Figure 2; median difference in time between first phase and second = 12.77; *p* = 0.023; 95% CI: 1.29, 1.44); however, mounting decreased when a novel female was placed in the test mouse's cage the following week (Supplementary Figure 2; median difference in time between first week and second = −19; *p* = 0.004; 95% CI: −31.89, −6.10).

### 3.3. NL3 Mice Are Aggressive towards Female Mice and Isolation Housing Reduces Incidence of Stalking

**Figure 4:**
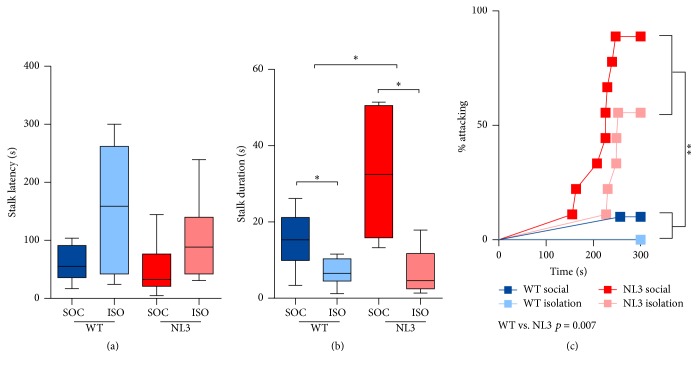
NL3 male mice show aggression towards female mice. (a) Latency to stalk female mouse, (b) duration of stalking female, and (c) percentage of male mice attacking female. Values are an average of 4 tests (2 phases, 2 weeks) and data in (a-b) are displayed as boxplots with median plus the 25th and 75th percentiles. Whiskers represent the minimum and maximum values. SOC = socially housed animals (WT: *n* = 10; NL3 = 10); ISO = isolation-housed (WT: *n* = 9; NL3 = 9) animals. ^*∗*^*p* < 0.05 and ^*∗∗*^*p* < 0.01.

NL3^R451C^ and WT mice were monitored for signs of aggression towards female mice during all phases of the test. While no genotype effect on stalking latency was seen (Figure 4(a)), NL3^R451C^ mice stalked female mice for a longer duration (Figure 4(b); median difference in time between WT and NL3 mice = 9.42; *p* = 0.038; 95% CI: 0.53, 18.30). Regardless of genotype, mice housed in isolation were less likely to stalk the female, showing higher latencies to stalk (Figure 4(b); hazard ratio of first stalk in social compared to isolation housing = 0.92; *p* = 0.021; 95% CI: 0.21, 0.88) and reduced duration of stalking (Figure 4(c); median difference in time between social and isolation housing = −5.14; *p* = 0.045; 95% CI: −10.16, −0.11). NL3^R451C^ mice were more likely to attack female mice, regardless of housing, exposure, or week of test (Figure 4(c); hazard ratio of first attack in WT compared to NL3 mice = 18.20; *p* = 0.007; 95% CI: 2.21, 149.67). With the exception of one WT mouse, only NL3^R451C^ mice attacked female mice. Attacks were brief and did not occur many times per mouse, limiting the analysis to latency.

**Figure 5:**
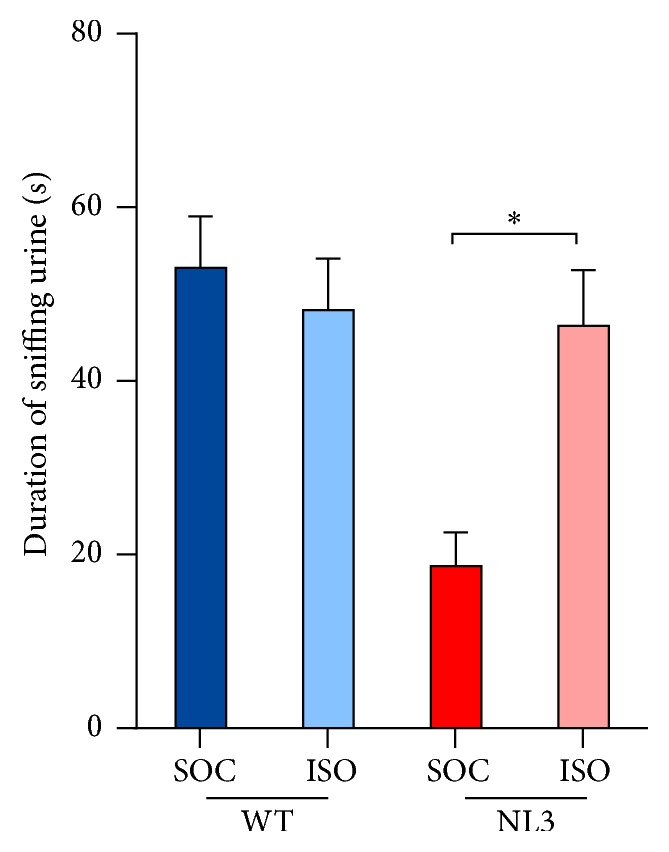
NL3 mice are less interested in female urine and social isolation increases interest to WT levels. Values are displayed as mean ± SEM. Asterisks represent a statistically significant difference between indicated groups. ^*∗*^*p* < 0.05. SOC = socially housed animals (WT: *n* = 10; NL3 = 10); ISO = isolation-housed (WT: *n* = 9; NL3 = 9) animals.

Time spent investigating estrus female urine was measured in a naïve cohort of mice and was used as an index of arousal and interest in the female. Housing specifically modulated NL3 mouse sniffing behavior (Figure 5; two-way ANOVA, genotype*∗*housing interaction:* F*_1,33_ = 11.264; *p* = 0.002). Pairwise comparisons indicated that socially housed NL3 mice investigated the stimulus for less time compared to their WT counterparts. Isolation housing increased investigation time in NL3 mice to levels comparable to WT mice.

## 4. Discussion

The present study identified heightened interest in mating and atypical aggressive behavior in NL3^R451C^ mice. NL3^R451C^ mice exhibit normal social interaction towards female mice, indicating that abnormal aggressive behavior is not due to altered motivation to engage in prosocial interactions. Isolation housing increased the time spent engaging in social interaction in all mice, in line with reports that social isolation increases motivation to engage in social communicative behaviors [40]. A selective effect of social isolation housing was seen on time spent investigating female pheromones in NL3^R451C^ mice. No difference in grooming was detected between NL3^R451C^ and WT mice, consistent with previous investigations into this behavior [33].

Heightened territorial aggression has previously been identified in NL3^R451C^ male mice utilizing the resident-intruder test whereby a male juvenile intruder mouse is introduced to the home cage of a test mouse [31]. The present study has shown that NL3^R451C^ mice also display aggressive behavior towards female mice. While aggression towards male mice is a robust, innate, social behavior to assist in the acquisition of social ranking and resources from the environment [41], aggression towards female mates is atypical. Studies employing similar paradigms to assess social interaction in male-female dyads have not shown aggression towards females in WT mice [42]. Rearing in social isolation leads to increased territorial aggression in adult mice [43, 44]. In the present study, socially housed animals did not show an increase in aggression. This discrepancy could be due to a number of factors. The atypical aggression seen in NL3^R451C^ mice may not be territorial in nature as the aggression is directed towards both males and females. Furthermore, testing was not conducted in the home cage of male test mice, reducing the likelihood and severity of aggression for those that showed the behavior. Using an assay designed to potentiate aggression would allow more thorough investigation of this atypical behavior in NL3^R451C^ mice and also in isolation-housed mice.

Abnormal aggression towards female mice has been linked to altered levels of brain serotonin, with mice deficient in brain tryptophan hydroxylase 2 exhibiting hyperaggressive behavior towards their female interaction partners [45]. The hyperaggression in NL3^R451C^ mice, previously identified towards male intruder mice, was mitigated following treatment with risperidone, an antagonist with high affinity for both serotonin and dopamine receptors [31]. Furthermore, overlapping neural circuits have been shown to control aggression and mating behavior in mice [46]. Specific activation of the ventral medial hypothalamus in male mice paired with a female, led to aggressive behavior in between bouts of mounting [35]. These findings provide a compelling reason to investigate both of these systems and brain regions in NL3^R451C^ mice and to interrogate their role in mediating aggressive behavior. Furthermore, it will be of interest to explore the efficacy of risperidone treatment to reduce the aggression observed in NL3^R451C^ mice towards female mice.

Since mice use pheromonal cues to identify individuals, we explored the possibility that pheromone detection may be altered in NL3^R451C^ mice and could underlie their atypical aggression towards females. Unlike WT mice, NL3^R451C^ mice showed reduced interest in exploring female urinary pheromones. Reduced interest could indicate that vomeronasal function may be compromised in NL3^R451C^ mice; however, we have previously shown no impairment in olfactory discrimination of social and nonsocial odorants, including female urine [31]. Furthermore, isolation housing increased time spent sniffing urine in NL3^R451C^ mice, indicating that the social housing was a likely factor in influencing interest in urinary pheromones. Social experience has been shown to modulate mating behavior, the production of vocalizations used during mating, and social interaction and response to pheromones [40, 47]. Mice normally live in large groups and exhibit social interactions that are dependent on the dynamics of multiple group members [48] and complex dominance hierarchies [49]. Further interrogation of dominance hierarchies in socially housed, mixed-genotype groups is therefore warranted.

In addition to aberrant aggression, NL3^R451C^ mice showed heightened interest in mating with female mice. Increased mating drive could underlie this phenotype and future studies of this mouse model should assay for blood testosterone concentration differences from baseline following exposure to a female. Given that NL3 knockout mice have been reported to show reduced vocalizations during contact with a female mouse [50], investigation of social communication during mating in NL3^R451C^ mice is also warranted.

This study identified heightened interest in mating and atypical aggressive behavior towards female mice in NL3^R451C^ mice. This is the first investigation of social interactions in male-female dyads in NL3^R451C^ mice and contributes to the full characterization of altered social behavior in this mouse model of ASD. Further investigation into the overlapping neural substrates underlying mating and aggressive behavior in the NL3^R451C^ mouse may shed light into the role of Neuroligin-3 in mediating complex social behavior in mice. NL3^R451C^ mice provide a very useful tool to model circuitry underlying abnormal social behavior in ASD.

## Supplementary Material

Supplementary Figure 1: High concordance of behaviors scored by an independent observer. a) bouts and b) duration of all behaviors present in 5 trials selected at random. Supplementary Figure 2: Duration of mounting increased in all mice when they were exposed to the same female after a brief period of separation (phase); however, this decreased when a novel female was placed in the test mouse's cage the following week (week 2). Data is displayed as boxplots with median plus the 25th and 75th percentiles. Whiskers represent the minimum and maximum values. SOC = socially-housed animals (WT: n=10; NL3=10); ISO = isolation-housed (WT: n=9; NL3=9) animals.

## Abbreviations

NL3: Neuroligin-3
R451C: Arginine to cysteine residue 451 substitution
WT: Wild-type.
