# Supplementary figures and images for: Social Isolation Alters Social and Mating Behavior in the R451C Neuroligin Mouse Model of Autism
Source: Neural Plast. 2017 Jan 31;2017:8361290. doi: 10.1155/2017/8361290 (PMC5307131; doi:10.1155/2017/8361290)

**a**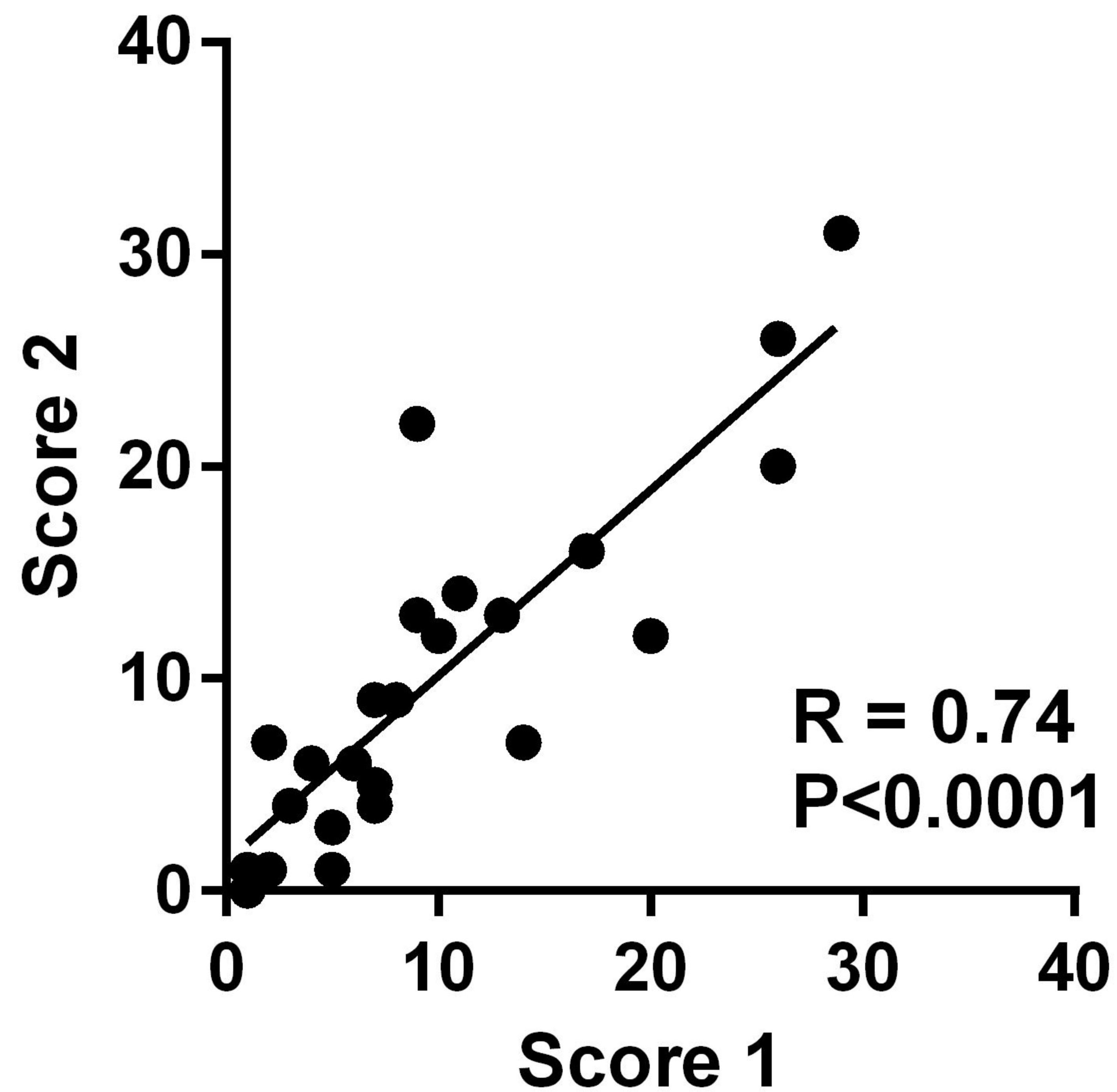**b**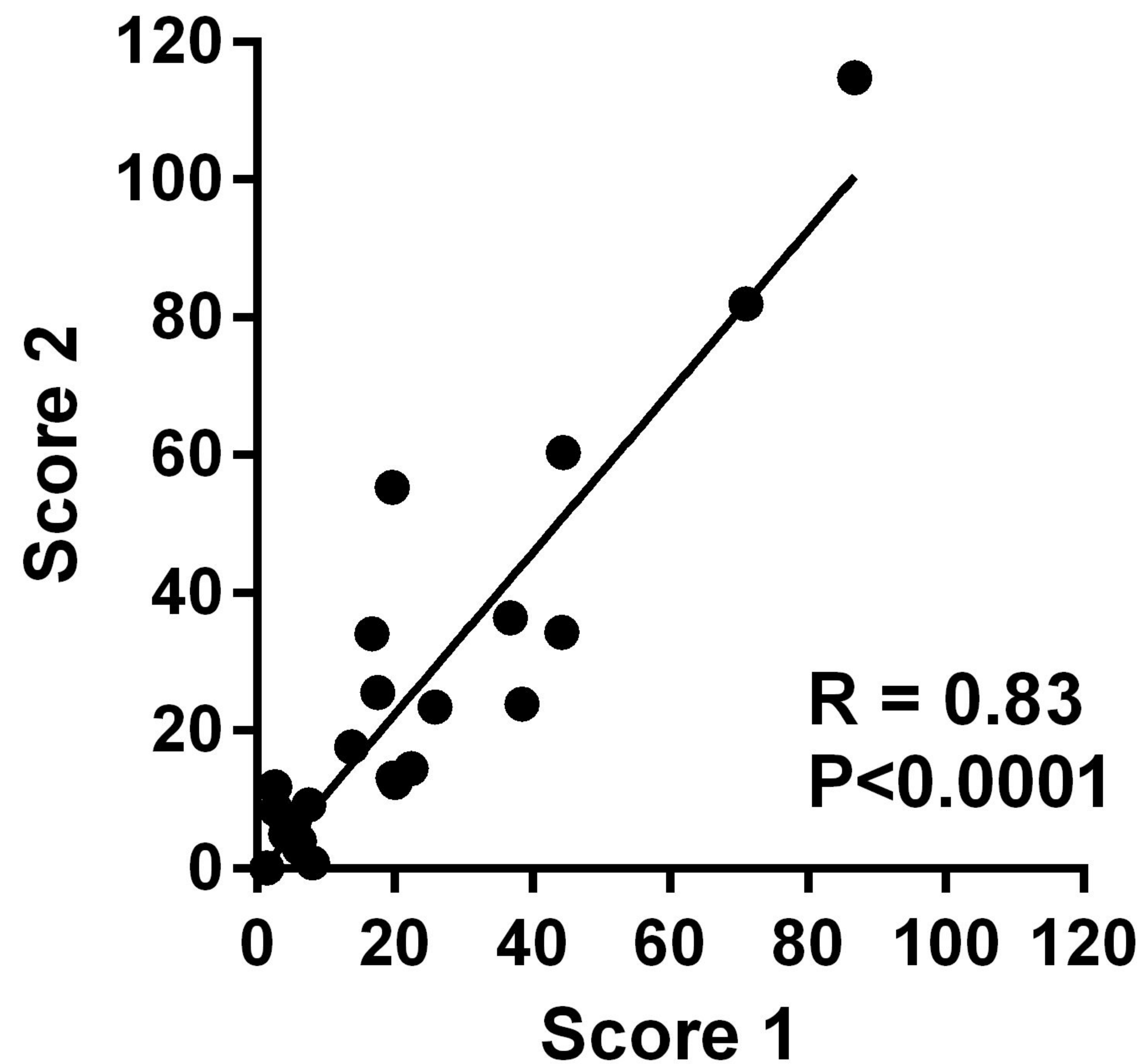

Supplement: Supplementary file 1 — Supplementary Figure 1: High concordance of behaviors scored by an independent observer. a) bouts and b) duration of all behaviors present in 5 trials selected at random. Supplementary Figure 2: Duration of mounting increased in all mice when they were exposed to the same female after a brief period of separation (phase); however, this decreased when a novel female was placed in the test mouse's cage the following week (week 2). Data is displayed as boxplots with median plus the 25th and 75th percentiles. Whiskers represent the minimum and maximum values. SOC = socially-housed animals (WT: n=10; NL3=10); ISO = isolation-housed (WT: n=9; NL3=9) animals. [file 8361290.f1.pdf]

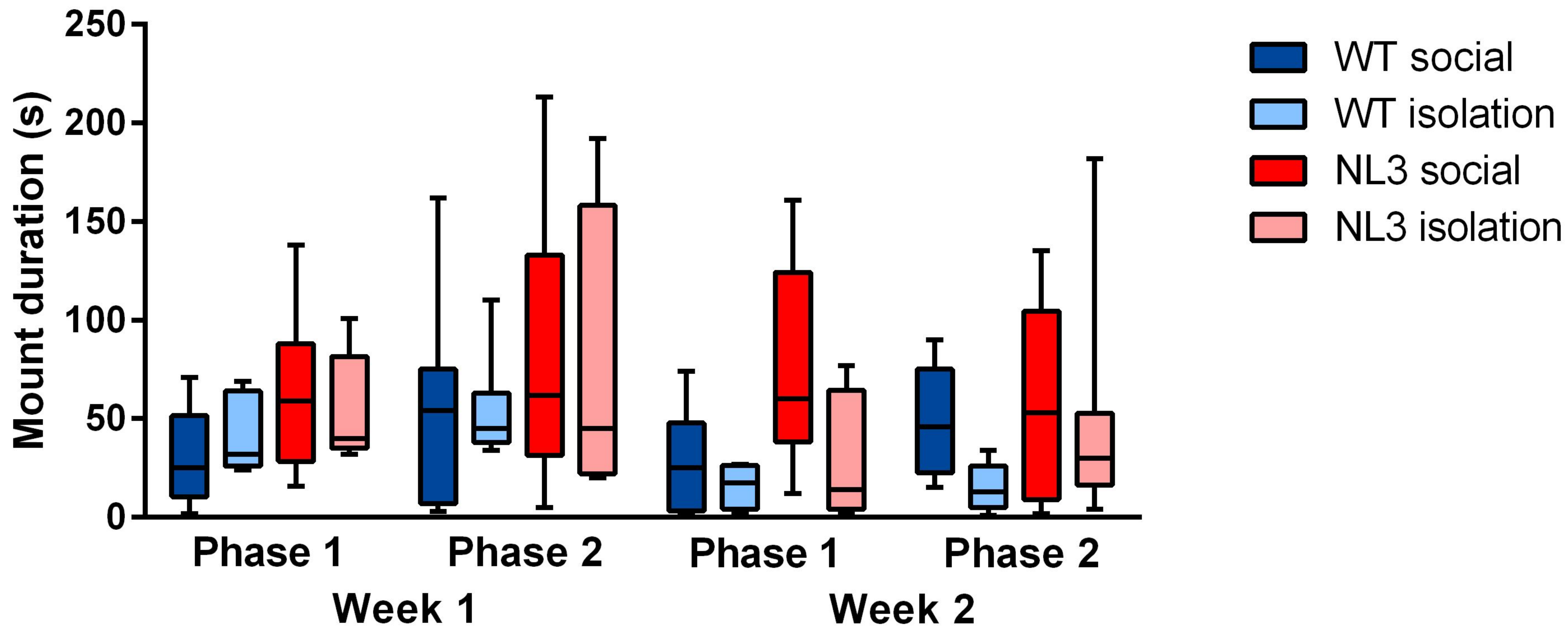

Supplement: Supplementary file 2 [file 8361290.f2.pdf]
